# Pathogens and Carcinogenesis: A Review

**DOI:** 10.3390/biology10060533

**Published:** 2021-06-15

**Authors:** Muhammad Nur Adam Hatta, Ezanee Azlina Mohamad Hanif, Siok-Fong Chin, Hui-min Neoh

**Affiliations:** UKM Medical Molecular Biology Institute (UMBI), Universiti Kebangsaan Malaysia, Jalan Ya’acob Latiff, Cheras, Kuala Lumpur 56000, Malaysia; P98904@siswa.ukm.edu.my (M.N.A.H.); ezanee.azlina.mohamad.hanif@ppukm.ukm.edu.my (E.A.M.H.); chinsiokfong@ppukm.ukm.edu.my (S.-F.C.)

**Keywords:** infections, pathogens, carcinogenesis

## Abstract

**Simple Summary:**

An increasing number of cancer cases has been reported throughout the years. Most cancers are linked to unhealthy lifestyles and genetic inheritance. Nevertheless, unknown to many, infection from microorganisms (bacteria, viruses, fungi) and sometimes, parasites, can also lead to cancer development. For these cancers, the infection may inflict mechanical injury on host cells, whilst gene products or protein secretion from the microorganism further alters host cell activity, leading to abnormal cell development and growth. Due to the cancer-causing characteristic of these microorganisms, they have been classified as definite biological agents that cause cancer. This review describes the cancer development process caused by some of these microorganisms and highlights strategies to prevent or treat the associated cancers.

**Abstract:**

Cancer is a global health problem associated with genetics and unhealthy lifestyles. Increasingly, pathogenic infections have also been identified as contributors to human cancer initiation and progression. Most pathogens (bacteria, viruses, fungi, and parasites) associated with human cancers are categorized as Group I human carcinogens by the International Agency for Research on Cancer, IARC. These pathogens cause carcinogenesis via three known mechanisms: persistent infection that cause inflammation and DNA damage, initiation of oncogene expression, and immunosuppression activity of the host. In this review, we discuss the carcinogenesis mechanism of ten pathogens, their implications, and some future considerations for better management of the disease. The pathogens and cancers described are *Helicobacter pylori* (gastric cancer), Epstein-Barr virus (gastric cancer and lymphoma), Hepatitis B and C viruses (liver cancer), *Aspergillus spp*. (liver cancer), *Opisthorchis viverrine* (bile duct cancer), *Clonorchis sinensis* (bile duct cancer), *Fusobacterium nucleatum* (colorectal cancer), *Schistosoma haematobium* (bladder cancer); Human Papillomavirus (cervical cancer), and Kaposi’s Sarcoma Herpes Virus (Kaposi’s sarcoma).

## 1. Introduction

Cancer is a disease in which cells divide in an uncontrolled manner and have the ability to invade nearby tissues. The disease is a significant cause of morbidity and mortality. From the latest annual cancer case report of 2020, the World Health Organization (WHO) estimated more than 19.2 million new cases being diagnosed and 9.9 million mortalities from cancer [1]. Carcinogenesis and the progression of cancer are usually undetectable externally; many cancers, such as pancreatic and colorectal cancers (CRC) are undiagnosed until they reach a later stage [2,3]. Risk factors associated with lifestyle, diet, and genetic predisposition have been identified to contribute to carcinogenesis [4]. Increasingly, infections caused by pathogenic microorganisms and parasites have also been linked to cancer [5,6,7,8,9].

**Figure 1:**
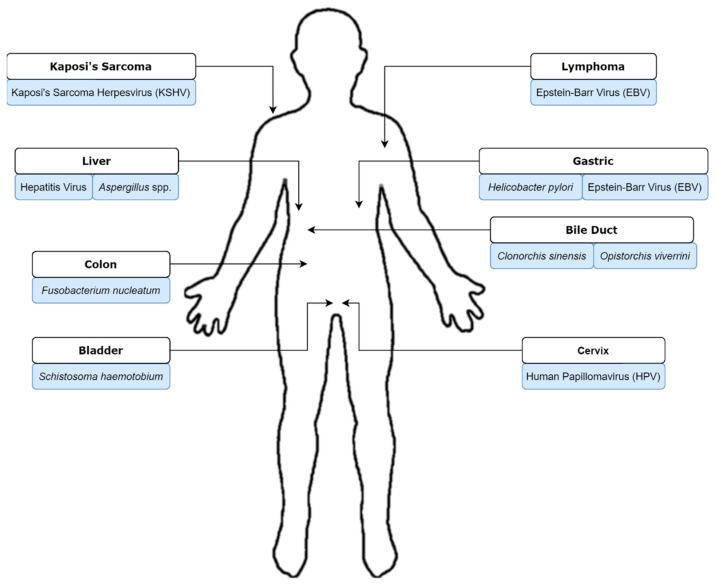
Tropism of pathogens associated with human cancer development described in this review.

Microorganisms have been primarily studied for their roles in causing infection. However, since the early 20th century, the association of some infectious agents with cancer has been reported [10]. Beginning in the 1900s with a report by Askanazy, the link between *Opisthorchis felineus* infection and liver cancer, in addition to the discovery of bladder cancer-causing Bilharzia infections (schistosomiasis), have been highlighted [11]. Subsequently, the remarkable finding of oncolytic viruses was reported by Peyton Rous in 1911 in an avian model [12]. Fifty years later, Anthony Epstein proposed and proved the association of the Epstein-Barr virus (EBV with Burkitt lymphoma [13,14]). Infectious agents cause carcinogenesis largely via three mechanisms [15]. The first mechanism is via persistent infection which gives rise to inflammation and cell damage, where accumulative cell damage leads to mutations and carcinogenesis. Alternatively, infectious agents might cause the expression of oncogenic genes of their host and lead to cancer. In the third scenario, immunosuppression caused by infectious agents leads to carcinogenesis in the host via immunologic recognition disruption [15]. Carcinogenesis tropism is observed in cancers caused by infectious agents, whereby different agents are associated with different types of cancer (Figure 1). This review will describe ten infectious agents whose roles have been confirmed in carcinogenesis.

## 2. Gastric Cancer

Gastric cancer (GC) ranked fifth in worldwide cancer incidence (1,089,103 new cases) and third in cancer-related mortality (768,793 mortalities) in the year 2020 [1]. Risk factors for GC include lifestyle modifiers such as dietary habits and smoking, family history, and socioeconomic status [16,17,18]. In addition, infection with *Helicobacter pylori* and EBV have also been reported to be risk factors for the occurrence of GC [19]. *H. pylori* infection is attributed to almost all GC cases; EBV contributes to less than 10% of incidences [20,21].

### 2.1. Helicobacter pylori

*Helicobacter pylori* is a Gram-negative microaerophilic bacterium that could be isolated from the upper gastrointestinal tract of more than 50% of the population [22]. It can be transmitted through saliva, vomit material, and feces [23]. The bacteria’s chemotaxis properties allow it to detect pH changes in the stomach and subsequently burrow into the epithelial layer to escape the acidic mucosal lining [24]. In addition, *H. pylori* secrete urease which breaks down gastric urea to produce carbon dioxide and ammonia, neutralizing the acidic pH of the environment [25]. While these properties allow the bacteria to survive in the stomach and duodenum, the production of ammonia is toxic to host epithelial cells, whereby long-term colonization of *H. pylori* causes inflammation and results in chronic gastritis [26,27].

For hosts who carry the *cag* pathogenicity island (PAI)-positive *H. pylori*, secreted CagA disrupts cellular processes and host cell gene transcription via tyrosine phosphorylation of SHP-2 and kinases, causing neoplastic morphological changes and cell proliferation [28,29,30]. At the same time, the type IV secretion system expressed by *cag* PAI injects bacterial peptidoglycan into gastric epithelial cells, stimulating cytokine expression and further inflammation [31]. In addition, 50% of all *H. pylori* strains secrete VacA, a virulence factor that induces epithelial cell vacuolization and inhibits T-cell activation and proliferation, aiding the bacteria’s gastric colonization and leading to peptic ulceration [29,32]. Subsequently, if lifestyle or genetic risk factors are present, for hosts who are low acid producers, gastric cancer might occur, while high acid producers might develop duodenal cancer [33]. Association of *H. pylori* and gastric inflammation has been reported since 1982, and the bacteria was later categorized as a group I (definite) carcinogen by the IARC in 1994 [34,35].

### 2.2. Epstein-Barr Virus (EBV)

The Epstein-Barr virus (*Human herpesvirus 4*) is usually associated with Burkitt’s lymphoma [14] and is the etiological agent of infectious mononucleosis. It is a capsulated DNA virus and spreads via body fluids such as saliva and genital secretions. It has been reported that most people will be infected with the virus at some point in their lives. After infection, the virus may remain dormant in the B cells of its immunocompetent host, until it is reactivated [36]. Besides lymphoma, EBV infection has also been associated with the occurrence of GC [37]. About 9% of GCs reported an EBV etiology, where meta-analyses on EBV-associated gastric cancers (EBVaGC) showed, intriguingly, slightly higher prevalence in young males of American and European populations [21,38] and a lower incidence in China [39].

While *H. pylori* infection is mostly associated with tumors in the gastric antrum, EBVaGCs are usually located at non-antrum sites of the stomach [40]. Compared to *H. pylori*-associated gastric cancer in which carcinogenesis is caused by the bacteria’s toxin secretion and ammonia production, EBVaGCs are associated with host genome methylation via EBV modulation [41,42,43]. In EBVaGC, the virus is orally ingested and reaches gastric epithelial cells via saliva carriage. The virus enters epithelial cells via the host cell receptors with B cell mediation, and is subsequently assembled into circular mini chromosomes (“episomes”) [44,45].

Once latent infection is established, viral transcripts and proteins contribute towards carcinogenesis in the stomach [45]. Viral non-coding RNAs, EBER-1 and -2, promote tumor cell proliferation and migration, while the protein EBV-determined nuclear antigen-1 (EBNA1) induces reactive oxygen species (ROS) accumulation and at the same time impairs the host response towards DNA damage [46,47,48,49]. Importantly, EBV latent membrane protein 2A (LMP2A) induces epigenetic changes to the host genome via methylation of CpG islands, inactivating tumor suppressor genes such as PTEN and tumor-associated antigens [41,50]. In fact, host genome methylation is a common characteristic of EBVaGC, in which besides PTEN, promoter hypermethylation of CDH1, p14ARF, p15, p16INK4a, and p73 tumor suppressor genes has also been associated with EBV infection [43]. In addition to host genome methylation, viral genome methylation further allows the pathogen to escape host immune detection. Recent research shows the involvement of EBV miRNAs, where the non-coding RNAs have been reported to upregulate cancer cell proliferation, inhibit apoptosis, and suppress interferons (IFN) signaling [51,52,53].

## 3. Liver Cancer

Hepatocellular carcinoma (HCC), commonly known as primary liver cancer has a survival rate of 6 to 20 months, and is one of the cancers with high mortality rates [54,55] with 830,180 recorded mortalities and 905,677 incidents in 2020 [1]. The disease occurs most often in individuals who have a history of chronic liver diseases such as cirrhosis [56,57]. Alcohol consumption, smoking, and metabolic conditions such as obesity and type 2 diabetes are HCC co-clinical factors. In addition, chronic infection by the hepatitis virus and consumption of food contaminated with aflatoxin from the *Aspergillus* fungi confers a high risk towards the development of HCC [58]. All the above risk factors subject the liver to a state of chronic inflammation, with ongoing cycles of oxidative stress, DNA damage, hepatocyte turnover, and fibrosis [59].

### 3.1. Hepatitis Virus

There are five hepatitis viruses (A–E); two of them, hepatitis B (HBV) and C (HCV), are associated with HCC [56]. Despite their tropism for the liver, HBV and HCV are from different families, namely, *Hepadnaviridae* for HBV and *Flaviviridae* for HCV. The HBV genome consists of double-stranded DNA with reverse transcriptase, while HCV is a single-strand RNA virus. These two viruses are transmitted through blood. Nevertheless, HBV can also spread via body fluids during sexual intercourse or vertical mother-to-infant transmission [60]. 

Epidemiological studies suggest chronic HBV infection as the main risk factor in HCC development [61,62,63], where low copy numbers of the virions are sufficient to initiate infection [64]. Once it enters the host, the virus makes its way to the liver, binds to hepatocytes via the NTCP (sodium taurocholate co-transporting polypeptide) receptor, enters the cell via endocytosis, and proceeds to the nucleus [65]. Viral DNA transcription and protein translation are then initiated [63]. During active infection, the newly generated viral DNA will be integrated into the host genome, leading to chromosomal instability (CIN), insertional mutagenesis, and cis-activation of tumor-associated genes. Interestingly, no consistent singular target gene for HBV DNA integration has been identified; though pathways associated with AKT activation, mitotic cell cycle, AXIN1, and DNA imprinting have been reported to be dysregulated by the infection [59]. In addition, binding of the Hepatitis B X protein (HBx), to the host genome changes the expression of miRNAs and further disrupts histone methyltransferases activity, leading to cell expression pattern changes in HCC pathophysiology [66,67]. In addition, although the full extent of Hepatitis D virus (HDV)-associated HCC pathogenesis remains to be investigated, studies so far have found that co-infection of the virus with HBV will increase hepatocyte necro-inflammation, leading to cirrhosis and HCC [68,69]. 

In chronic HBV infection, the host experiences phases of “immune tolerance,” “immune reactive,” “inactive carrier,” “chronic hepatitis,” and “HBV surface antigen-negative” [70]. The risk for HCC is higher at both “immune reactive” and “chronic hepatitis” phases. During the “immune reactive” phase, the virus infects hepatocytes and integrates into host DNA. Subsequently, during “chronic hepatitis,” HBV replication is lowered, allowing viral mutants to escape host immune response. Nevertheless, this will still drive inflammation and hepatitis progression in the host via continuous activation of impaired anti-viral immune response, which in turn exacerbates inflammation and hepatocyte turnover, causing clonal expansion of premalignant cells containing HBV-integrated host DNA and HCC [71]. 

Compared to HBV infection, HCV does not integrate into the host genome. There are two phases of HCV infection: acute and chronic [59]. The risk for HCC increases during the chronic phase, and may increase as much as 17-fold [72]. The virus is transcribed once it reaches and enters the host’s hepatocytes [73,74]. HCV viral factors are then implicated in the interference of a variety of molecular pathways, including cell metabolism, genetic repair, apoptosis, and induction of ROS activity [75]. 

Progression of HCV infection induces metabolic reprogramming of hepatocytes, causing hepatosteatosis where there is a deposition of excessive triglycerides in cells [76]. Viral phosphoprotein NS5A further activates the PI3K/AKT signaling pathway, which is integral to HCC development. Viral proteins and their genome block tumor suppressors such as p53 and the epidermal growth factor receptor (EGFR), and induces ROS activity via the mediation of NADPH oxidase-1 and -4 (NOX) [77]. Chronic HCV infection also triggers both innate and adaptive immunity of the host, where inflammatory cytokines including tumor necrosis factors (TNF), interleukin (IL), and lymphotoxins (LT) are increased due to activation of inflammatory pathways such as NF-κB [78]. All the above produces a carcinogenic microenvironment promoting genetic instability and the development of hepatic stellate cells (HSCs) [79,80]. Epithelial to mesenchymal trans-differentiation (EMT) changes in HSCs are regulated by TGF-β growth factor then further contributes to carcinogenesis [81].

### 3.2. Aspergillus spp.

Two species from the *Aspergillus* fungus, namely *Aspergillus flavus* and *Aspergillus parasiticus* produce the genotoxic compound aflatoxin, which can be found in improperly stored food crops, such as rice, wheat, millet, corn, and peanuts [82,83,84]. The toxin, when ingested via food supplies, may cause acute aflatoxin poisoning and lead to abdominal pain and vomiting. Serious cases of acute exposure have been reported to cause pulmonary edema, fatty liver, liver necrosis, and even death [85]. Intriguingly, the toxin has a tropism for the liver, where chronic exposure to the toxin has been proven to increase HCC risk in humans and many species of animals. Epidemiologically, aflatoxin exposure is linked to HCC in many West African countries due to inappropriate post-harvest processing; in addition, developing countries have a higher incidence rate, where low-income populations sometimes resort to long-term consumption of moldy food produce to avoid starvation [86,87,88].

Although there are four aflatoxins (AFB1, AFB2, AFG1, and AFG2), AFB1 is the most common and has been strongly associated with HCC [85]. Once ingested, AFB1 will find its way to the liver and is subsequently activated by microsomal enzymes, forming DNA adducts of trans-8, 9-dihydro-8- (N7-guanyl)-9-hydroxyaflatoxin B1 (AFB1-N7-dG), and trans-8, 9-dihydro-8- (2, 6-diamino-4-oxo-3, 4-dihydropyrimid-5-yl-formamido) -9-hydroxy aflatoxin B1 (AFB1-Fapy-dG) [89]. AFB1-N7-dG has been reported to cause G > T mutagenesis [90], while AFB1-Fapy-dG may cause all G > T, G > A, G > C and single nucleotide deletions in p53 [91,92]. In addition to the formation of DNA adducts, AFB1 has also been found to cause mutations (AGG > AGT) at codon 249 of p53, leading to arginine substitution with serine (R249S) [92,93]. Hosts without proficient nucleotide or base excision repair mechanisms, together with by-pass by the error-prone DNA polymerase ζ during DNA replication will lead to clonal expansion of hepatocytes with p53 allelic deletions. Coupled with chronic inflammation due to HBV or HCV infections, chronic hepatitis and/or liver cirrhosis will occur, with HCC as a sequela.

## 4. Bile Duct Cancer

Risk factors for bile duct cancer or also known as cholangiocarcinoma (CCA) include older age, smoking, chronic liver disease, and primary sclerosing cholangitis (PSC) [94]. The cancer remains rare in the western hemisphere. Nevertheless, in southeast Asia, the prevalence is higher, where the disease is usually caused by helminth (parasite) infection. *Opisthorchis viverrini* and *Clonorchis sinensis*, two species of liver flukes, are the causative pathogens via food contamination [95]. Duration and intensity of the infection, host and liver parasite genetics, diet, and environmental exposure determine if the infection will lead to CCA [96]. Even though the etiology of liver fluke-associated CCA is known, many patients present at the later stages of III and IV with unresectable tumors, rendering the disease with poor clinical outcomes [95]. Both parasites are classified as group I human carcinogens by the IARC in 2012. 

### 4.1. Opisthorchis viverrini

*Opisthorchisviverrini* was first discovered in Southeast Asia in 1886 in a fish by the parasitologist Jules Poirier [97]; it is prevalent in Thailand, Laos, Vietnam, and Cambodia. It is a monoecious hermaphrodite [98] and requires three different hosts (two intermediate and one definitive) to complete its life cycle. *O. viverrini* miracidia larvae infect freshwater snails (*Bithynia* spp.) and grow into sporocysts in snail tissues. These sporocysts become cercaria larvae, escape from snail tissues, and migrate towards fish, their second intermediate host. The flukes will then develop into metacercaria in the flesh of the fish. Ingestion of raw, contaminated fish frees *O. viverrini* into their final host, where they migrate towards the biliary tree and dominate the bile duct [99,100]. The flukes then cause CCA via three mechanisms: mechanical and chronic injury to biliary epithelial cells, immunologic inflammation via release of reactive oxygen intermediates and nitric oxide, and host cell proliferation via parasite secretion products. In chronic infection, these will cause DNA damage to the host cells and lead to tumorigenesis [101].

The parasites establish localization in biliary cells via securing their oral and ventral suckers into the cell epithelia. This damages the host bile ducts, with the development of ulcers as the infection progresses. The presence of the parasite and its secretion products induce an immune reaction from its host, prompting the release of pro-inflammatory cytokines mediated by toll-like receptor (TLR) signaling. Secretion products from the fluke are usually proteins for nutrient digestion and host tissue invasion. Proteomic investigations identified one of these proteins as Ov-GRN-1, a granulin-like parasite growth factor that has been shown to cause aberrant growth of the biliary cells [102]. In addition, thioredoxin and thioredoxin peroxidase are produced by this parasite to induce an anti-apoptotic mechanism [103]. Intriguingly, secretion products from *O. viverrini* contribute towards wound healing processes in the host to counter mechanical injuries caused by its suckers on the cells. However, as the parasites feed continuously, complete recovery of the biliary epithelial is not achieved. 

Repeated cycles of cell division during incomplete wound healing subsequently leads to DNA damage and genomic instability of the host. Whole exome sequencing studies in *O. viverrini*-associated CCA patients revealed mutations in genes of canonical carcinogenesis pathways such as TP53, KRAS, and SMAD4 [104]. Mutations in genes associated specifically with CCA were also identified, these include RNF43, PEG3, BAP1, ARID1A, MLL3, IDH1/2, GNAS, and ROBO2 [105]. Host genomic instability, together with the presence of other carcinogenetic factors such as dietary nitrosamines (found in salted or fermented fish, a common dish in southeast Asia), conduce a microenvironment that is favorable for malignancies [101,105,106]. Of note, besides dietary nitrosamine, *O. viverrini*-associated CCA cases carrying active *H. pylori* infections have been observed, and hamster infection models showed an obligatory mutual relationship of the fluke with the carcinogenic bacteria [107].

### 4.2. Clonorchis sinensis

*Clonorchis sinensis,* the Chinese liver fluke, is mainly found in East Asian countries such as China, Taiwan, Korea, and Northern Vietnam [108]. Like *O. viverrini*, the parasite is digenetic, with snails and cyprinid fish as intermediate hosts. It shares a similar mechanism of infection and carcinogenesis as *O. viverrini*—by causing mechanical damage and chemical irritation to its host. 

In addition to mechanical damage, feeding of the frequently propagating parasites at the bile ducts serves as mechanical obstruction, leading to metaplasia of the biliary epithelial cells. These cells will transform into mucin-producing cells that produce excretory-secretory products (ESPs) and mucus in the bile [109]. At the same time, the presence of the parasite at the biliary tree is recognized by host TLR-2 and -4, resulting in the production of inflammatory cytokines and chemokines. These peptides were originally intended for fluke elimination, however, they now contribute towards disease progression, causing toxicity towards the host, and cholangiocyte damage [110]. 

Production of ESPs from the parasite was found to induce metabolic oxidative stress [103,110], activating inflammatory mediators such as NADPH oxidase, nitric oxide synthase, lipoxygenase, cyclooxygenase, along with xanthine oxidase to generate free radicals, exacerbating the inflammatory response mediated by NF-κB [111,112]. In particular, the production of nitric oxide leads to host DNA damage by DNA repair inhibition and cyclooxygenase stimulation [113,114]. Generated free radicals will subsequently cause lipid peroxidation (LPO), a process that increases cell proliferation and deactivates cell apoptosis. In addition, ESPs might trigger host transcriptome, proteome, and miRNA expression changes via processes such as histone modification and mini-chromosome maintenance (MCM) regulation [115]. In chronic infection, all the above factors contribute to host genome instability and increased fragility to carcinogens, which, coupled with environmental risk factors such as the consumption of dietary nitrosamines, will lead to CCA.

## 5. Colorectal Cancer

Colorectal cancer (CRC) has affected more than 1.9 million people worldwide in the year 2020 with 935,173 mortalities [1]. While diet such as frequent red meat consumption and family history were reported to be associated with CRC, gut microbiome dysbiosis was recently identified as a risk factor for CRC [116]. Several bacteria, such as *Bacteroides fragilis*, *Streptococcus gallolyticus*, *Streptococcus bovis*, and *Fusobacterium nucleatum* have been reported to have a higher abundance in CRC patients. Among them, *F. nucleatum* has been suggested as a potential microbial carcinogen that initiates the development of CRC [117,118].

### Fusobacterium nucleatum

*Fusobacterium nucleatum* are anaerobic Gram-negative bacteria that were first isolated from the oral cavity. With the advent of gut microbiome profiling, it was discovered that the bacteria can also colonize human intestines [119]; nevertheless, the movement of *F. nucleatum* from the mouth to colon remains unclear. In 2013, Kostic et al. proved that infection by *F. nucleatum* increases tumor cell multiplicity and recruits tumor-infiltrating myeloid cells in an in vivo model. Accordingly, carcinogenic properties of the bacteria were then reported, where the bacteria were found to enhance the proliferation of normal human colon cells, subsequently triggering the epithelial-mesenchymal transition pathway [120].

*Fusobacterium nucleatum* contributes toward the development of CRC via a few pathways. The bacteria attach and invade colon endothelial cells via the FadA adhesion protein. This causes the secretion of cytokines (IL-6, 8, 10, 18; TNF-α) and the expression of NF-κB, creating a pro-inflammatory environment in the colon [121]. At the same time, macrophage infiltration and methylation of the cyclin-dependent kinase inhibitor 2A, CDKN2A [122] occurs in the tumor microenvironment. In addition, FadA binding of the host cell E-cadherin receptor activates β-catenin signaling. This promotes tumor cell proliferation via increased expression of oncogenes of the Wnt pathway and their transcription factors [118]. Chronic infection activates the *p38* gene which is crucial in the production of matrix metalloproteinase (MMP)-1, -9, and -13 for invasion as well as metastasis properties [123]. Besides FadA, *F. nucleatum* harbors another virulence factor, Fap2, which binds to TIGIT, an inhibitory receptor on T cells and natural killer cells, protecting tumor cells from the host immune system. Indeed, the bacteria were found to inhibit human T-cell responses toward mitogens and antigens (immunosuppressive activities) [124], most probably via blockage of the cell cycle mid-G1 phase [125].

Of note, in the case of CRC, cross-talk between microbial species might be important in causing cancer. Other than *F. nucleatum*, bacteria such as *Peptostreptococcus stomatis*, *Parvimonas micra*, and *Akkermansia muciniphila* have been found to be over-represented in the gut mucosa of CRC patients [126,127,128,129]. The exact role of these bacteria in CRC pathogenesis, however, remains to be investigated.

## 6. Bladder Cancer

In 2020, more than 573,000 cases of urinary bladder carcinoma were newly reported worldwide, followed by 212,536 mortalities [1]. The cancer includes urothelial carcinoma, squamous cell carcinoma, and adenocarcinoma; some can involve more than one cell type. In many parts of the world, squamous cell carcinoma can be caused by chronic irritation to the bladder as a result of prolonged urinary catheter usage. Nevertheless, in the Middle East and Africa regions, the cancer is associated with urogenital schistosomiasis caused by *Schistosoma haematobium* parasites [130].

### Schistosoma haematobium

Urogenital schistosomiasis is a medical condition caused by *S. haematobium* infections. This infection leads to chronic inflammation and the presence of blood in the urine—hematuria [131], a condition associated with the development of bladder cancer. *S. haematobium* was first discovered in the 1850s in Cairo, Egypt, and carcinogenic properties of the parasite were later reported in the late 1880s [132,133]. Compared to other parasites, this trematode lives in pairs (male and female) and undergoes sexual reproduction during their life cycle. The free-swimming parasite could be acquired from freshwater environments, where it enters human hosts via skin penetration. This process is mediated by the secretion of proteolytic enzymes [134]. Following this, the cercaria larvae will migrate to its favorable site of infection (uterus, bladder, and prostate) for reproduction. Eggs of *S. haematobium* can be traced from urine samples of infected patients; eggs that are deposited in the bladder wall will cause damage and inflammation to the bladder lumen [135], increasing the risk of bladder cancer.

In chronic infection, the stuck eggs induce a granulomatous host T helper 2 (TH2) immune response due to prolonged inflammation, and urinary bladder irritation [136,137]. H03-H-IPSE, a major ortholog of the interleukin-4-inducing principle (IPSE) protein secreted by *S. haematobium* eggs, was found to induce urothelial cell proliferation in mouse models with nuclear localization, driving the cells towards the S-phase of the cell cycle. The protein also induces bladder angiogenesis [135] and allows the eggs to escape the host immune system. All the above leads to urothelial hyperplasia, a pre-cancerous lesion. In addition to its eggs, adult parasites have also been shown in xenogeneic animal models to increase cell proliferation and migration, as well as to decrease apoptosis [135]. Nevertheless, a single exposure to a parasite antigen will not contribute to tumorigenesis, suggesting the need for chronic infection to cause cancer in hosts. Interestingly, bacterial and parasite co-infection in females was reported to increase bladder cancer risk, though the mechanism of carcinogenesis is still unclear [130]. Indeed, urine microbiome dysbiosis in urogenital schistosomiasis has also been reported in bladder cancer and other pathologies of the organ [138].

In hosts, metabolism of parasite molecules, such as catechol estrogens and guanine-derived oxidation products will lead to genotoxic effects such as mutation, DNA strand breakage, and sister chromatid exchanges induced by the hydroxyl radical from inflammatory cells [130,139,140]. Chromosomes 1, 3, 5, 6, 7, 8, 9, 11, 14, 15, 17, 18, and Y are the most frequent site with abnormalities observed in bladder cancer development [141,142], where deletion in chromosome 9 has been associated with *S. haematobium* infections, leading to the loss of important protein function crucial in activating p53 and retinoblastoma (Rb) pathways and anti-apoptotic programs [143,144]. Urogenital schistosomiasis will also cause molecular perturbation via overexpression of the fibroblast growth factor receptor protein 3, causing the aggressive proliferation of cells [143]. In addition, mutations in KRAS have also been observed [145]. 

Due to its propensity to cause genetic and epigenetic changes that lead to cell hyperplasia and cancer, *S. haematobium* has been classified as a group I definitive biological carcinogenic agent by the IARC in 2012 [146]. Moreover, exposure to pro-carcinogenic factors such as smoking and N-nitroso compounds (either from diet, or from dysbiosis of urine microbiome in schistosomiasis) will increase bladder cancer risk in urogenital schistosomiasis [130,147,148].

## 7. Cervical Cancer

Cervical cancer is mostly diagnosed among women at the age of 35 to 44 years old. It is the fourth most frequent cancer reported globally with 604,127 cases in 2020 [1]. The burden of cervical cancer faced by low- and middle-income countries is significantly greater than in high-income countries, and contributed to 341,831 deaths in 2020 [1,149]. Interestingly, cervical cancer is usually caused by infectious agents, namely the human papillomavirus (HPV). Co-infections by *Chlamydia trachomatis* can initiate chronic infection on the endocervical cells at the transformation zone, which exposes the cells to oncogenic HPV infections [150].

### Human Papillomavirus (HPV)

*Human Papillomavirus* is considered the principal etiological agent [151] and classified as a carcinogen for cervical cancer. There are currently more than 200 HPV subtypes, where they are classified as group I, IIA, IIB, and III carcinogens by the IARC. Amongst the HPV subtypes, HPV 16 and HPV 18 have been described as potential human carcinogens since 1983 [152]. Risk factors for HPV-associated cervical cancer include smoking and sexual exposure with multiple sexual partners, where these partners in turn, also have multiple partners [153]. 

All HPV subtypes are naked (non-enveloped) viruses with a small diameter (~55 nm) and a circular double-stranded DNA genome [154]. The virus is epitheliotrophic, where, after sexual contact (vaginal, anal, or oral) with an infected person, it will bind to heparin sulfate proteoglycans on the cervical basal basement membrane through breaks in the epithelium [155,156]. The virus will then be uncoated and directly transported to the host nucleus for replication, where early proteins (E1–E7) and late capsid proteins (L1 and L2) will be synthesized [157]. Episomal copies of the HPV genome will remain inside the infected cells [158,159]. E1 and E2 will be expressed for the production of virions to invade other neighboring cells while viral capsids aid viral movement inside the host cell during infection [160]. The oncogenic properties of HPV are mostly conferred via E6 and E7. 

During chronic HPV infection, integration of the virus genome inside the host cell might occur, where cells will transition into cervical intraepithelial neoplasia grade I (CIN-1). E6 and E7 proteins interfere with cell proliferation activity controlled by p53 and pRb, [161] and also inhibit cell cycle checkpoint control via cyclin-dependent kinase (CDK) inhibitors (p21, p27, p16) [162]. These molecular events facilitate anti-apoptosis activity, disrupt the DNA repair mechanism, and deregulate cell cycle control, driving differentiating cells into the S-phase and rapid proliferation. 

Lesions from CIN-1 may develop into CIN-2 or -3 in the space of 2 to 3 years. During this development, abnormal proliferation of cervical cells will occur, driven by the upregulation of genomics signatures such as those from the Kinesin family member (KIF23), Integrin subunit alpha V (ITGAV), CDKN2A, and Centromere Protein E (CENPE). Particularly, during the later CIN stages, proteins such as BUB1 mitotic checkpoint serine/threonine kinase B (BUB1B), mitotic arrest-deficient 2 (MAD2L1), checkpoint kinase 1 (CHEK1), cyclins, and proteins involved in the cell division cycle are found to be highly regulated [163]. From CIN-3, further failure to prohibit cell proliferation will lead to invasive carcinoma.

## 8. Kaposi’s Sarcoma

Kaposi’s sarcoma (KS) is a rare cancer lesion that appears on the skin, mouth, and nose lining, lymph nodes, or other vital organs. Globally, KS contributed 34,370 new cases with 15,084 mortalities in 2020 [1]. The lesions are usually purple in color, and consist of lymphatic endothelial cells or their precursors, which will develop into clonal metastases of spindle cells in the advanced stage [164]. KS cases can be divided into four subtypes: (1) epidemic, (2) classic, (3) endemic, and (4) iatrogenic [165]. Epidemic KS is associated with the HIV/AIDS epidemic [166]. Classic and endemic KS refer respectively to cases that occurred in elderly southern European and Middle Eastern males, and children in equatorial, eastern, and southern African countries. Iatrogenic KS mostly occurs in organ transplant patients receiving immunosuppressive therapy. The causative pathogen in KS has been identified as Kaposi’s sarcoma herpesvirus (KSHV), also referred to as human herpesvirus 8 (HHV8) [167]. The virus was originally thought to co-evolve with the human population; nevertheless, studies identified higher seroprevalence amongst sub-Saharan Africans and Mediterranean populations compared to northern Europeans, northern Americans, and Asians [168]. KSHV mother-to-child transmission or transmission between siblings and playmates is common in endemic countries. On the other hand, viral transmission has been reported to be more common in homosexual men with multiple partners in non-endemic countries [169].

### Kaposi’s Sarcoma Herpesvirus (KSHV)/Human Herpesvirus 8 (HHV8)

Following the discovery of its genome sequences in biopsies, KSHV was identified as the causative agent for KS (IARC Class I carcinogen) [167]. The virus is a gammaherpesvirus with five major subtypes (A, A5, B, C, and D). Interestingly, the virus encodes proteins homologous to its human host, such as cyclin, viral FLICE inhibitory protein (vFLIP), B cell lymphoma 2 (BCL-2), IL-6, interferon regulatory factors, and chemokines, where viral cyclin and vFLIP promote the proliferation of infected tumor cells during latent infection [164]. Besides KS, KSHV is also associated with Castleman’s disease and primary effusion lymphoma, both neoplasms of the lymphatic system. 

Routes of KSHV transmission are still not elucidated, though it has been postulated that the virus is transmitted via saliva, and upon host entry, infects a variety of cells, such as endothelial cells, epithelial cells, and fibroblasts [170]. The virus can also infect cells of the immune system including monocytes, B cells, and dendritic cells [171]. Once it enters the target cell, KSHV will be uncoated, with its genome circularizing to become an episome in the nucleus. It will then either become latent (causing lifelong infection) or undergo cycles of lytic reactivation. Proteins from both stages can contribute to tumorigenesis. vFLIP and viral miRNAs from the latent stage stimulate pathways such as NF-κB to increase host cell survival and prevent apoptosis, while promoting vascular proliferation and leading to inflammation [172,173]. These changes can also be caused by lytic proteins such as KSHV vIL-6 via the expression of vascular endothelial growth factor (VEGF) and platelet-derived growth factor (PDGF) [174,175,176]. 

Of note, although KSHV oncogenic proteins have been shown to prevent apoptosis in experimental models, complete tumorigenesis events require the existence of co-factors, such as intake of immunosuppression drugs by the host, or the presence of HIV virus co-infection. HIV-1 encodes a transcriptional trans-activator, Tat, which increases KSHV infectivity [177] and induces apoptosis of CD4^+^ T cells [178]. Another HIV protein, Nef, regulates the AKT signaling pathway, facilitating angiogenesis and KSHV oncogenesis by boosting levels of vIL-6 [179,180] and other cytokines (IFN-γ, TNF-α, IL-1), causing reactivation of the KSHV life cycle via the JAK/STAT pathway [180,181].

## 9. Lymphoma

Lymphoma refers to blood malignancies that arise from lymphocytes (B cells, T cells, and natural killer cells), with most lymphomas from B cell origin (90%) [182]. The cancer is broadly divided into Hodgkin (10%) and non-Hodgkin (90%) lymphoma. The total cases reported for lymphoma in 2020 is worrying, with 627,439 newly diagnosed cases and 283,169 fatalities [1]. Patients usually experience enlarged lymph nodes, night sweats, weight loss, and tiredness. Oncogenic mutation(s) in lymphocytes followed by clonal propagation has been reported in lymphomas [183], where age, male gender, and an impaired immune system have been identified as risk factors [184,185]. In addition, infection by pathogens such as human T-cell leukemia virus, HHV8, *H. pylori*, HCV, and EBV has been reported to be associated with lymphoma. Among these, EBV has been shown to cause a variety of lymphomas, including Burkitt’s lymphoma (BL) and Hodgkin lymphoma (HL) [186].

### Epstein-Barr Virus (EBV)

Epstein-Barr virus-associated lymphomas usually occur during childhood (asymptomatic) with a progression towards infectious mononucleosis during adolescence. Primary infection occurs via the oral route, leading to viral entry and replication in the oral mucosal epithelium and B cells, where it enters the lytic phase. EBV may also enter pharyngeal lymphoid tissues, where the virus will switch to its latent phase [187] as episomes, and lifelong infections occur in the presence of latent membrane proteins (LMP) expression [11,15]. Infected cells carry EBNA, LMP, EBV-encoded RNA (EBER), and EBV miRNAs [188,189]. 

Cases of EBV-associated BL are mostly reported in regions where malaria is hyperendemic [190]. Malaria infection activates the host immune response to produce B cells and translocation of c-*MYC*, leading to increased B cell proliferation [191]. Together with mutations in ARF-MDM2-p53 pathways, p53-dependent apoptosis events are prevented [188]. EBNA-1 possesses oncogenic properties which cause high levels of ROS production and increased NOX2 catalytic subunits of the NADPH oxidase, leading to genetic instability [192]. Lymphocyte immortalization is further controlled by EBNA-2 nuclear protein via the expression of cellular proteins such as EBV receptor/CR2 (CD21) [193]. During latent infection, EBV has been reported to convert human B cells to become lymphoblastic cell lines (latency III infection) via aggressive lymphoblast proliferation [194,195]. 

In contrast, EBV-associated HL is characterized by the formation of multinucleated Reed-Sternberg (HRS) cells or mononucleated Hodgkin cells derived from B lymphocytes [188]. Most HRS cells possess a defective rearrangement of their B cell surface, disrupting normal cell signaling functions [196]. LMP-1 and LMP-2 are highly expressed in HRS, where these proteins complement cell surface defects and play a vital role in their survival [197,198]. In addition, genetic mutations such as translocation of *CIITA* (*MHC2TA*) have been observed in 15% of HL cases [199]. These mutations cause further disruptions in lymph node structure and cause the infiltration of non-neoplastic inflammatory-cells, increasing the release of various inflammatory cytokines (IL-1, IL-6, TGF-β, and TNFα) [188].

## 10. Implication of Infectious Agents in Carcinogenesis and Future Considerations

It is now evident that some cancers, even though not categorized as infectious diseases, can be caused or further exacerbated by infectious agents via various carcinogenesis pathways (Table 1). This discovery has a few implications. Firstly, some of these cancers may be prevented with vaccinations or other public health measures that prevent the human host from coming into contact with cancer-causing pathogens. In addition, periodical screening or infection surveillance in patients and intake of drugs, such as antimicrobials may help to prevent pre-cancerous lesions from worsening.

Vaccinations against HBV and HPV infections have contributed to the lowering of hepatitis (and subsequently, HCC) and cervical cancer incidences. The implementation of infant HBV vaccination programs in many countries since 1995 has significantly reduced the incidence of pediatric hepatitis and primary liver cancer [200]. On the other hand, as cervical cancer is mostly a sequela of HPV infection, it is projected that high vaccine coverage will enable the global elimination of this cancer [201]. Other public health measures have also proved important in controlling infections by cancer-associated pathogens and thereby lowering cancer incidence. Establishing good regulating systems of staple dietary foods and improving storage conditions of crops will reduce *Aspergillus* transmission and GC [84,85,86]. Snail control to prevent schistosomiasis infections has been shown to be effective in reducing parasite infection [202,203]. In Thailand, behavioral-psycho-social interventions are being carried out to increase public awareness of the risks of raw fish consumption to reduce fluke infections and CCA [204].

Pathogen biomarker-based screening for patients has been integral in the detection and monitoring of infected patients, paving the way for early intervention to prevent tumorigenesis. Prior to HPV vaccination, Papanicolaou (Pap) smears with HPV tests allow for the detection of precancerous lesions and contribute to the lessening of cervical cancer burden. Indeed, Pap smear screening is still important even after HPV vaccination and is recommended to be carried out every 3 or 5 years, according to risk factors of the population [205]. Endoscopic surveillance involves the monitoring of the gastric environment to detect cellular changes, due to *H. pylori* and EBV infections [20,21], where the treatment of patients using antibiotics (*H. pylori*) to lower GC risk [206], or immunotherapy using checkpoint inhibitors (EBV) for treatment of GC [45] (which is currently being tested in clinical trials), can ensue for positive cases. Besides antibiotics, antimicrobials such as antivirals have been found to prevent pre-malignant stages of virus-associated cancers, such as IFN-free anti-viral therapies for HCV [207], zidovudine and valganciclovir for KSHV [208], and anti-worm medication such as praziquantel or albendazole to eliminate flukes and schistosomiasis [209].

Moving forward, multiomics studies into pathogens coupled with molecular editing will further contribute towards the discovery of better vaccination and treatment targets, along with improved strategies for pathogen detection, control, and elimination. This will enhance the prevention, early detection, and treatment of associated cancers. In addition, treatment using oncolytic viruses that infect and kill targeted cancer cells will also be a new paradigm in cancer therapy. 

## 11. Conclusions

Infection by some pathogens may trigger carcinogenesis pathways that lead to cancer in susceptible individuals. Identification of pathogens that can function as human carcinogens, understanding how exposure to these pathogens occurs, and the subsequent carcinogenic mechanisms they trigger will be important. Knowledge in these areas will provide useful clues for successful pathogen-associated cancer management, control, and ultimately, prevention.

## Figures and Tables

**Table 1:** Carcinogenesis mechanism and prevention/treatment strategies for infection-associated cancers.

Pathogen	Type of Cancer	Carcinogenesis Mechanism	Prevention/Treatment
*Helicobacter pylori*	Gastric	Disruption of the cellular mechanism via tyrosine phosphorylation of SHP-2 and kinases mediated by CagA secretion Induction of epithelial vacuolization, T-cell activation, and proliferation by VacA	Endoscopic surveillance Antibiotics (amoxicillin, clarithromycin, metronidazole, tetracycline, tinidazole)
Epstein-Barr Virus (EBV)	Gastric	Viral integration into the host genome mediated by B cell Cell proliferation and migration promotion by Viral EBER-1 and -2 transcript ROS accumulation and subsequent DNA modification via viral EBNA1 induction CpG island methylation, tumor suppressor genes, and tumor-associated antigens inactivation via viral LMP2A Cancer proliferation, apoptosis inhibition, and IFN signaling suppression via EBV miRNAs	Immunotherapy (checkpoint inhibitors) Antivirals (acyclovir, ganciclocvir, valgancyclovir, omaciclovir, valomaciclovir, maribavir, cidofovir, thymidine derivatives)
Hepatitis B Virus (HBV)	Liver	Chromosomal instability, mutagenesis, and cis-activation of tumor-associated genes due to viral-host genome integration miRNA expression alteration, histone methyltransferases activity, and cell expressions pattern dysregulation by HBx protein	Vaccination Antiviral therapy (lamivudine, adefovir, entecavir, telbivudine, tenofovir, emtricitabine, standard, and PEG-IFN)
Hepatitis C Virus (HCV)	Liver	Interference in metabolic reprogramming of hepatocytes Activation of PI3K/AKT pathway by NS5A phosphoprotein Blockage of tumor suppressor gene activity by HCV proteins Activation of ROS by NOX-1 and -4 Activation of inflammatory pathways and cytokines	Reducing the risk of exposure (single-use needles for intravenous drug injection, protection during sexual intercourse) Antiviral therapy (simeprevir, sofosbuvir, ledipasvir-sofosbuvir, ombitasvir-paritaprevir-ritonavir-dasabuvir, sofosbuvir-velpatasavir, sofosbuvir-velpatasvir-voxilaprevir, glecaprevir-pibrentasvir, ribavarin)
*Aspergillus* spp.	Liver	Formation of DNA adducts via activation of microsomal enzymes by aflatoxins Clonal expansion of hepatocytes	Food safety and storage management (high temperature, gamma rays) Detoxification agent (bacteria: *Pleurotus eryngii*; plant extract: *Adhatoda vasica* Nees) Chemical treatment (novasil clay mineral, chlorophyll)
*Opisthorchis viverrini*	Bile Duct	Mechanical and chronic injury of biliary epithelial cells due to parasite attachment Inflammation due to parasite product secretion Abnormal growth of biliary cells caused by Ov-GRN-1 protein Anti-apoptotic mechanism induction via thioredoxin and thioredoxin peroxidase production Incomplete wound healing due to DNA damage and chromosomal instability Mutagenesis in canonical carcinogenesis pathways	Proper food preparation (avoid consumption of raw fish) Antiparasitic (praziquantel, albendazole)
*Clonorchis sinensis*	Bile Duct	Host biliary cell metaplasia due to chronic mechanical irritation by parasite attachment Oxidative stress, host transcriptome, proteome, and miRNA expression changes due to ESPs Host DNA damage by genotoxins Cell apoptosis deactivation and abnormal cholangiocytes proliferation due to lipid peroxidation	Proper food preparation (avoid consumption of raw fish) Antiparasitic (praziquantel, albendazole)
*Fusobacterium nucleatum*	Colorectal	Cytokine production activation via FadA–E-cadherin adhesion Macrophage infiltration and CDKN2A methylation Cell proliferation activation via β-catenin and Wnt pathway upregulation Cancer cell invasion via MMP-1, -9, and -13 production Host immune system evasion via attachment of Fap2 to immune cells	Antibiotics (piperacillin, amoxicillin-clavulanate, clindamycin, imipenem, metronidazole) Chemotherapy (*COX-2* inhibitor, specific EP2 antagonist) Immunotherapy (anti-Fap2 antibody, CTLA-4, PD-1, miR-21 blockade, adoptive cell transfer)
*Schitosoma haematobium*	Bladder	Granulomatous host Th2 immune response induction via chronic egg deposition Urothelial cell proliferation and bladder angiogenesis by H03-H-IPSE protein Mutation, DNA damage, and sister chromatid exchanges via parasite metabolites Excessive cell proliferation due to FGFGR3 overexpression	Snail control Water treatment (chlorine) Antiparasitic (praziquantel, albendazole)
Human Papillomavirus (HPV)	Cervical	Tumor suppressor genes interference by E6 and E7 Cell cycle check point control inhibition Anti-apoptosis induction, DNA repair mechanism disruption, abnormal proliferation, cell cycle dysregulation KIF23, ITGAV, CDKN2A, CENPE, BUB1B, MAD2L1, CHEK1, cyclin, and cell cycle proteins upregulation at a late stage	Vaccination Protection during sexual intercourse Regular screening (Pap smears) Surgical procedure (cryotherapy, electrocautery, surgical removal, laser surgery)
Kaposi’s Sarcoma Herpesvirus (KSHV)	Kaposi’s Sarcoma	Proliferation of cancer cells by vFLIP Apoptosis prevention, vascular proliferation, and inflammation via vFLIP, vIL-6, and/or viral miRNAs	Reducing the risk of exposure (avoid unprotected sexual intercourse) HAART treatment (for HIV patients) Antiviral therapy (valgancilovir, foscarnet, zidovudine) Radiotherapy Immunotherapy Chemotherapy (liposomal anthracyclines) Inhibitor agent (mTOR inhibitor, proteasome inhibitor, paclitaxel, MMP inhibitor, anti-angiogenic agents)
Epstein-Barr Virus (EBV)	Lymphoma	**Burkitt’s Lymphoma** Apoptosis inhibition via p53 mutations Genetic instability due to ROS and NOX2 production via EBNA1 Lymphocyte immortalization regulation via EBNA2 B to lymphoblastic cell line generation due to chronic infection	Avoid body fluid transfer from infected patients Small molecule inhibitor (EBNA1 inhibitor, HDAC inhibitors, butyrate and GCV, bortezomib, CDKs inhibitors, PI3K inhibitors, BCL-2 inhibitors, mTOR inhibitors, ixazomib) Immunotherapy (immune checkpoint inhibitors) Cell therapy (monoclonal antibodies, T-cell therapy)
		**Hodgkin’s Lymphoma** Formation of multinucleated HRS cells from B lymphocytes Cell signal disruptions due to defective HRS cells Increased HRS cell survival due to LMP-1 and -2 expression Lymph node structure disruption and cytokine activity increment due to CIITA mutagenesis	

## Data Availability

Not applicable.
